# Editorial: Insights in vascular physiology: 2025

**DOI:** 10.3389/fphys.2026.1946734

**Published:** 2026-08-11

**Authors:** Luis A. Martinez-Lemus, Christopher Garland, Andrew P. Braun

**Affiliations:** 1 Department of Medical Pharmacology and Physiology, University of Missouri, Columbia, MO, United States; 2 The Vascular Pharmacology Group, Department of Pharmacology, University of Oxford, Oxford, United Kingdom; 3 Department of Physiology and Pharmacology, University of Calgary, Calgary, AB, Canada

**Keywords:** adipokine, endothelium, immune cell, mechanotransduction, pericyte, vascular smooth muscle

This Research Topic edition, “Insights in Vascular Physiology: 2025,” illustrates the complexity of vascular control and remodeling from complementary perspectives, including vascular smooth muscle, pericytes, lymphatic smooth muscle, endothelium, immune cells, and adipokine signaling. The contributions highlight how vascular function is shaped by mechanical forces, ion channel activity, calcium handling, sex-specific biology, metabolic stress, neuroimmune communication, and genetic variability.

Sun and Hill provide a conceptual framework for how vascular smooth muscle contraction may be organized across the arterial wall. They propose that myosin light chain phosphorylation, a fundamental determinant of smooth muscle force generation, peaks in the outer-layer smooth muscle cells, suggesting that wall tension and mechanotransduction may establish transmural gradients of contractile activation. This model also integrates mechanical inputs from the vessel wall with chemical signals from the endothelium, raising important questions about the mechanisms that control the phenotypic characteristics of mural cells across the vascular wall.

The article by Zhao and Lederer expands this mural cell perspective to pericytes in the heart. Using a perfused papillary muscle preparation that preserves physiological characteristics, the authors present a technical approach for high-resolution visualization of pericytes within the beating-heart microcirculation. Images reveal an elaborate pericyte network, including “bridging” pericytes with extensions that span capillary surfaces and contact cardiomyocytes. These findings suggest that cardiac pericytes may contribute not only to capillary support but also to intercellular signaling, mechanical coordination, and microvascular regulation in the myocardium.

Mohajeri et al. examine whether mechanical stimulation can restore contractility in aged vascular smooth muscle from resistance arteries. They show that a brief increase in intraluminal pressure can rescue contractile function in aged arteries. At the cellular level, aged smooth muscle cells respond to cyclic stretch and hydrostatic pressure by remodeling actin stress fibers and cell-matrix adhesions, expanding focal adhesion molecules. These findings imply that age-related vascular dysfunction may remain mechanically modifiable, and that the cytoskeleton and focal adhesions of smooth muscle represent key targets for preserving arterial function with aging.

Suarez et al. further refine the mechanisms of vasoconstriction by demonstrating sex-specific roles for T-type voltage-gated calcium channels in hypertension. In renal arteries from hypertensive rats, females exhibited enhanced phenylephrine-induced vasoconstriction and greater participation of T-type calcium channels. In contrast, males showed no corresponding increase in T-type channel contribution to contraction. These findings underscore that similar hypertensive phenotypes may arise through distinct mechanisms in males and females and support the goal of developing sex-informed precision medicine approaches to vascular therapy.

The study by Arriero-Carrillo et al. focuses on aldosterone-dependent remodeling of intracellular calcium handling in smooth muscle cells. By examining SERCA2a and SERCA2b expression and localization, the authors show that mineralocorticoid receptor activation increases both isoforms and redistributes them toward subplasmalemmal regions. This re-organization coincides with increased calcium spark frequency and calcium waves, suggesting that aldosterone modifies the capacity and spatial organization of calcium-handling processes. The work identifies SERCA activity as a potential mechanism regulating calcium handling microdomains in smooth muscle, thereby linking hyperaldosteronism to altered vascular function.

Castorena-Gonzalez et al. extend the theme of contractile regulation to lymphatic vessels. In collecting lymphatics from a rodent model of type 2 diabetes and obesity (db/db mouse), they demonstrate marked reductions in contraction amplitude, frequency, and calculated pump flow. Importantly, impaired lymphatic pumping was restored by KATP channel inhibition, while high glucose reproduced aspects of the dysfunction in control lymphatics. Resistance to high-glucose impairment in Kir6.1-deficient vessels further supports a role for KATP channel activation in metabolic stress-induced lymphatic dysfunction. This study broadens the perspective that metabolic syndrome impairs the lymphatic muscle pump, potentially contributing to dysfunction in tissue fluid homeostasis and immune cell trafficking.

The review by Albinsson et al. examines Hippo signaling and the transcriptional coactivators YAP and TAZ in vascular smooth muscle and endothelial cells. In smooth muscle cells, YAP and TAZ maintain contractile identity, and their loss impairs contractility and promotes vascular pathology, including hypotension and aneurysm formation. They emphasize that YAP/TAZ signaling has potentially beneficial roles in smooth muscle but pro-atherogenic effects on endothelium. This divergence illustrates the therapeutic challenge of selectively targeting mechanosensitive signaling pathways that affect multiple vascular cell types.

The endothelium-focused article from Pislaru and Haas emphasizes that endothelial cells are not a uniform inner lining of the vasculature but a highly specialized, context-sensitive cellular system. This review highlights sex as a major determinant of endothelial phenotype, summarizing evidence that male and female endothelial cells differ in nitric oxide signaling, metabolism, angiogenic potential, oxidative stress responses, inflammatory activation, and susceptibility to senescence. These sex-dependent characteristics are further modified by vascular bed location, age, hormonal environment, sex chromosome, epigenetic regulation, and disease state. This perspective is especially important for vascular physiology because endothelial dysfunction is a common feature of hypertension, atherosclerosis, metabolic syndrome, and aging. By framing endothelial heterogeneity through the lens of sex-specific biology, the review moves beyond treating sex as a simple variable to analyses of how sex-linked pathways shape endothelial function and vulnerability. When considered alongside studies on T-type calcium channels and Hippo-YAP/TAZ signaling, this contribution reinforces the importance of integrating sex and cell-specific context into experimental design and therapeutic development.

Endothelial mechanisms also intersect with several of the smooth muscle-focused articles in the Research Topic. The hypothesis introduced by Sun and Hill explicitly incorporates diffusion-limited gradients of endothelial relaxing factors as counter-regulators of smooth muscle myosin light chain phosphorylation, while Suarez et al. demonstrate that altered nitric oxide availability modifies calcium channel-dependent vasoconstriction in a sex-specific manner. These links illustrate how endothelial biology cannot be separated from smooth muscle function, as endothelial signals establish local environments that modulate contractility, tone, remodeling, and vascular resistance.

Two articles expand the Research Topic’s scope beyond traditional vascular wall cells to include immune and metabolic regulators of vascular physiology. Grisanti and Nekouian review adrenergic receptor signaling in immune cells and its relevance to cardiovascular disease. They highlight that the cardiovascular consequences of sustained sympathetic activation are not mediated solely through cardiomyocytes, smooth muscle cells, or endothelial cells. Immune cells also translate catecholamine signals into inflammatory, cytotoxic, or pro-resolving processes. By integrating receptor-subtype signaling with desensitization, trafficking, compartmentalized signaling, and noncanonical pathways, they stress that adrenergic regulation of immunity depends on immune lineage, disease stage, and receptor state, and may amplify vascular injury or support resolution and repair. This perspective linkage between neurohumoral activation and vascular pathophysiology challenges the development of adrenergic therapies to reduce inflammation without compromising host defense.

The original research article by Fróes de Castro et al. examines adiponectin in hypertensive disorders of pregnancy. Their study links circulating adiponectin levels to ADIPOQ polymorphisms and haplotypes, showing that genetic variation may influence both adiponectin concentrations and susceptibility to pregnancy-associated vascular disorders. The finding that adiponectin levels are higher in preeclampsia than in healthy pregnancy, and that specific ADIPOQ variants are associated with disease risk or protection, highlights the complex role of adipokines in vascular and placental physiology. This contribution further connects vascular dysfunction to metabolic signaling, genetic variability, and pregnancy-specific cardiovascular risk.

Taken together, the ten articles appearing in the “Insights in Vascular Physiology: 2025” Research Topic provide an overview of how vascular function is controlled across scales, from molecular signaling domains to intact tissue physiology and systemic diseases. The mural cell articles emphasize spatial regulation of contraction, mechanically induced remodeling, calcium handling, and metabolic impairment of contractile function. The endothelial-focused contributions highlight heterogeneity, sex-specific biology, and the need to interpret vascular responses in a cell- and context-dependent manner. The immune cell and adiponectin articles extend the discussion to neuroimmune and metabolic-genetic mechanisms that influence vascular disease risk. These contributions also point toward future directions. More work is needed to define how spatially organized signaling networks operate in intact vessels, how endothelial and smooth muscle mechanisms interact across vascular beds and form signaling corridors, and how sex, age, metabolic status, and immune phenotype modify physiological and therapeutic responses. The articles collectively demonstrate that progress in vascular physiology will depend on understanding not only individual pathways but also the interactions among mechanical, electrical, metabolic, inflammatory, and genetic signals that shape vascular function in health and disease.

